# Efficacy and safety of inetetamab-containing regimens in patients with HER2-positive metastatic breast cancer in first-line/second-line setting

**DOI:** 10.3389/fonc.2025.1564888

**Published:** 2025-05-29

**Authors:** Jian Zhang, Yuxin Mu, Hui Zhang, Chao Deng, Jiao Yang, Lu Gan, Qingmo Yang, Xuefeng Xu, Wanping Liang, Xiaowei Qi, Liang Xu

**Affiliations:** ^1^ Department of Medical Oncology, Fudan University Shanghai Cancer Center, Shanghai, China; ^2^ Phase I Clinical Trial Center, Fudan University Shanghai Cancer Center, Shanghai, China; ^3^ Department of Oncology, Shanghai Medical College, Fudan University, Shanghai, China; ^4^ Department of Breast Surgery, Fujian Provincial Hospital, Shengli Clinical Medical College, Fujian Medical University, Fuzhou, China; ^5^ Breast Center (Department of Internal Medicine), Chongqing University Three Gorges Hospital, Chongqing, China; ^6^ Department of Oncology, The First Affiliated Hospital of Xi’an Jiaotong University, Xi’an, China; ^7^ Departmen of Medical Oncology, The First Hospital of Chongqing Medical University Affiliated Hospital, Chongqing, China; ^8^ Department of Breast Surgery, The First Affiliated Hospital of Xiamen University, Xiamen, China; ^9^ Department of Breast Surgery, Zhangjiakou First Hospital, Zhangjiakou, China; ^10^ Department of Breast Surgery, The First Affiliated Hospital of Hebei Northern University, Zhangjiakou, China; ^11^ Department of Thyroid Breast Surgery, Southwest Hospital of Army Medical University, Chongqing, China; ^12^ Department of Medical Oncology, Anyang Cancer Hospital, Anyang, China

**Keywords:** HER2-positive metastatic breast cancer, inetetamab-containing regimens, pyrotinib, pertuzumab, chemotherapy, first-line/second-line therapy

## Abstract

**Background:**

Inetetamab is a novel recombinant humanized anti-Human epidermal growth factor receptor 2 (HER2) monoclonal antibody. This real-world retrospective study assessed the efficacy and safety of inetetamab-containing regimens in first-line/second-line treatment of HER2-positive metastatic breast cancer (MBC).

**Methods:**

This study retrospectively recruited HER2-positive MBC patients who received inetetamab- containing regimens from June 2020 to May 2023. The outcomes included progression-free survival (PFS), objective response rate (ORR), and disease control rate (DCR).

**Results:**

A total of 329 patients were enrolled and included in the efficacy analysis. The most frequently used treatment strategy was contained inetetamab plus pyrotinib (205/329, 62.3%). Patients treated with first-line regimens benefited the most, with a median PFS of 15.0 versus (vs.) 10.0 months (first-line- vs. second-line inetetamab plus pyrotinib, *p <*0.001), 19.0 vs. 17.0 months (first-line- vs. second-line inetetamab plus pertuzumab, *p*=0.096), and 13.0 vs. not reached months (first-line- vs. second-line inetetamab plus chemotherapy, *p*=0.229). The complete response (CR) was observed in 16 (4.9%) patients of all cohort, with the ORR was 51.1% (95% confidence interval [CI], 45.7%-56.4%), and the DCR was 96.4% (95% CI, 93.7%-97.9%). The grade 3 or higher adverse events (AEs) were observed in 29.5% of the whole study cohort. Diarrhea (39.2%), white blood cell count decreased (33.0%), and myelosuppression (18.6%) as the most frequent ones.

**Conclusions:**

Following the first- and second-line of treatment, inetetamab- containing combinations demonstrated promising clinical activity and a manageable safety profile in patients with HER2-positive MBC, especially in the first-line treatment.

## 1 Introduction

Breast cancer had the highest incidence and mortality among females with malignant tumors worldwide (1), and the incidence and mortality were ranked first and fourth in China, respectively (2). Human epidermal growth factor receptor 2 (HER2)-positive breast cancer accounts for 20%-25% of all breast cancers and has a high invasive potential and poor outcome before the emergence of anti-HER2 therapy (3–5). Trastuzumab single or dual HER2-targeted therapy with pertuzumab is the standard treatment for patients with early HER2-positive, locally advanced, and advanced metastatic breast cancer (MBC) (6, 7). Beyond standard treatment, inetetamab, also known as Cipterbin^®^, a trastuzumab biosimilar, is a monoclonal antibody binding to domain IV of HER2 receptor (8). Inetetamab with amino acid modification of the Fc region has a more potent antibody-dependent cellular cytotoxicity (ADCC) effect than trastuzumab, which plays a key role in the antitumor activity of anti-HER2 monoclonal antibodies (9). Since it has shown good efficacy and safety in previous clinical studies (8, 10), it has been included in the treatment guidelines for breast cancer in China and has been recommended for treating advanced HER2-positive breast cancer.

Besides, it should be also noted that inetetamab-containing regimens were also frequently used in Chinese clinical practice, with tyrosine kinase inhibitors (TKIs), pertuzumab, and chemotherapy being common combining agents (8, 10, 11). Pyrotinib is an irreversible TKI of HER1, HER2, and HER4 that promotes cellular apoptosis and inhibits the proliferation of cancer cells (12). A retrospective study by Liu et al. reported that inetetamab combined with pyrotinib and vinorelbine might be the most effective treatment regimen for HER2-positive MBC, with a median PFS of 8.2 months (13). In the successive phase II and III (PHOEBE) studies, pyrotinib showed good antitumor effects with acceptable tolerability, with a median PFS of pyrotinib plus capecitabine was 12.5 months (14, 15). Overall, although there is a wide array of first-line and second-line regimens in current clinical use, determining the optimal choice for medications in clinical practice remains a concern. Although several real-world studies have been reported (11, 13, 16), therapeutic data still need to be supplemented, such as the comparison of inetetamab’s efficacy in combination with different regimens.

Here, we retrospectively reviewed patient data in a real-world setting to provide a more comprehensive and in-depth understanding of the clinical sequencing of medications and the selection of treatment regimens. The primary objective of this study was to evaluate the efficacy of inetetamab-containing regimens for HER2-positive MBC patients, and the secondary objective was to assess the safety profile.

## 2 Methods

### 2.1 Study design and patients

This retrospective, multicenter, real-world study enrolled patients between June 2020 and May 2023 at sixteen sites across China. Patients aged ≥18 years with pathologically confirmed HER2-positive MBC, which was defined as 3+ for immunohistochemical (IHC) analysis or 2+ for gene amplification by fluorescence *in situ* hybridization (FISH) staining of tumor tissue samples were enrolled in this study. Eligible patients received an inetetamab-containing regimen as first- or second-line treatments and had adequate organ functions and left ventricular ejection fraction (LVEF) ≥50%. Patients who had known previous or active allergies to the ingredients of the investigational drug, severe heart disease, and mental illness or psychotropic substance abuse were ineligible for this study. Pregnant or lactating patients, as well as patients with childbearing potential who did not use contraception if sexually active, were also excluded.

The Ethics Committee and Institutional Review Board of Affiliated Cancer Hospital of Fudan University (No. 1612167-18) and other participating centers approved this study. All investigations were conducted in accordance with the Declaration of Helsinki. Informed consent from patients was waived by the ethics committee based on this study being retrospective in nature.

### 2.2 Data collection and outcomes

Clinical data of patients, including demographic and baseline characteristics and prior treatment characteristics, was retrieved from the medical records. Outcomes included progression-free survival (PFS, defined as the time from the first administration of medication to the first recorded disease progression or death from any cause), objective response rate (ORR, calculated as the percentage of participants with complete response [CR] or partial response [PR]), and disease control rate (DCR, referred to the proportion of patients achieved CR, PR or stable disease [SD]). Safety was gauged by adverse event (AE), which was graded using the National Cancer Institute Common Terminology Criteria (NCI CTCAE) version 5.0. Tumor was assessed based on the Response Evaluation Criteria in Solid Tumors (RECIST) version 1.1.

### 2.3 Statistical analysis

Pearson’s χ2 test or Fisher’s exact test was used to compare the categorical variables. PFS was estimated using the Kaplan-Meier method and compared using the log-rank test for between-group differences. Multivariate analysis was performed using a Cox hazard proportion model to determine the survival difference and was reported with a hazard ratio (HR) and 95% confidence interval (CI). All statistical tests were two-sided, and a *p*-value <0.05 was considered significant. All analyses were conducted with obtained data, using SAS software version 9.4 (SAS Institute, Cary, NC, USA).

## 3 Results

### 3.1 Baseline characteristics

A total of 329 patients were enrolled and the baseline characteristics were presented in **Table 1**. The median age of the enrolled patients at diagnosis was 52 (range, 26-83) years. One hundred and nineteen (36.2%) patients were pre-menstrual, and the median DFI was 31.2 (range, 0-266.2) months. Most patients (312/329, 94.8%) had Eastern Cooperative Oncology Group performance status (ECOG PS) of 0–1 and 175 (53.2%) patients were diagnosed with hormone receptor (HR)-positive. The HER2 expression was IHC3+ in 133 (40.4%) patients, IHC2+ and FISH+ in 196 (59.6%) patients. The majority had lung metastasis (151/329, 45.9%) and bone metastasis (122/329, 37.1%). Regarding inetetamab-containing regimens, patients were treated with inetetamab combined with pyrotinib plus chemotherapy (defined as inetetamab plus pyrotinib group; 205/329, 62.3%), followed by inetetamab combined with pertuzumab plus chemotherapy (defined as inetetamab plus pertuzumab group; 54/329, 16.4%), inetetamab plus chemotherapy (defined as inetetamab plus chemotherapy group; 70/329, 21.3%). The median drug exposure time was 381 (range, 1-969) days and 18.1 (range, 0-46.1) cycles.

**Table 1:** Baseline characteristics of study patients.

Characteristics	Patients, No (%) n=329
Age, median (range)-years	52.0 (26.0-83.0)
Menstrual status	
Pre	119 (36.2)
Post	210 (63.8)
DFI, median (range)-months	31.2 (0-266.2)
ECOG PS	
0-1	312 (94.8)
2	17 (5.2)
HER2 expression	
IHC2+ and FISH+	196 (59.6)
IHC3+	133 (40.4)
Hormone-receptor status	
ER and/or PR positive	175 (53.2)
ER and PR negative	154 (46.8)
Metastatic organ site	
Liver	76 (23.1)
Lung	151 (45.9)
Brain	59 (17.9)
Bone	122 (37.1)
Previous anti-HER2 treatment	
Trastuzumab	196 (59.6)
Pertuzumab	69 (21.0)
Pyrotinib	29 (8.9)
Lapatinib	2 (0.6)
T-DM1	1 (0.3)
Treatment lines for Previous trastuzumab treatment	
(Neo)-adjuvant setting/First-line	196 (59.6)
No	133 (40.4)
Inetetamab-containing treatment regimen	
First-line	233 (70.8)
Second-line	96 (29.2)
Inetetamab + Pertuzumab	
Capecitabine	5 (1.5)
Albumin-bound paclitaxel	36 (10.9)
Vinorelbine	7 (2.1)
Docetaxel	6 (1.8)
Inetetamab + Pyrotinib	
Eribulin	14 (4.3)
Albumin-bound paclitaxel	43 (13.1)
Vinorelbine	113 (34.3)
Capecitabine	35 (10.6)
Inetetamab + Chemotherapy	
Eribulin	5 (1.5)
Albumin-bound paclitaxel	36 (10.9)
Docetaxel	8 (2.4)
Gemcitabine	1 (0.3)
Capecitabine	17 (5.2)
Utidelone	1 (0.3)
Taxane	2 (0.6)

Data are expressed as n (%), n (%; 95%CI).

No, number; DFI, Disease-Free Interval; ECOG PS, Eastern Cooperative Oncology Group performance status; IHC, Immunohistochemistry; FISH, Fluorescence in Situ Hybridization; ER, Estrogen receptor; PR, Progesterone receptor; HER2, Human Epidermal Growth Factor Receptor 2; TNM, Tumor, Node, Metastasis; T-DM1, trastuzumab emtansine.

### 3.2 Efficacy

**Figure 1:**
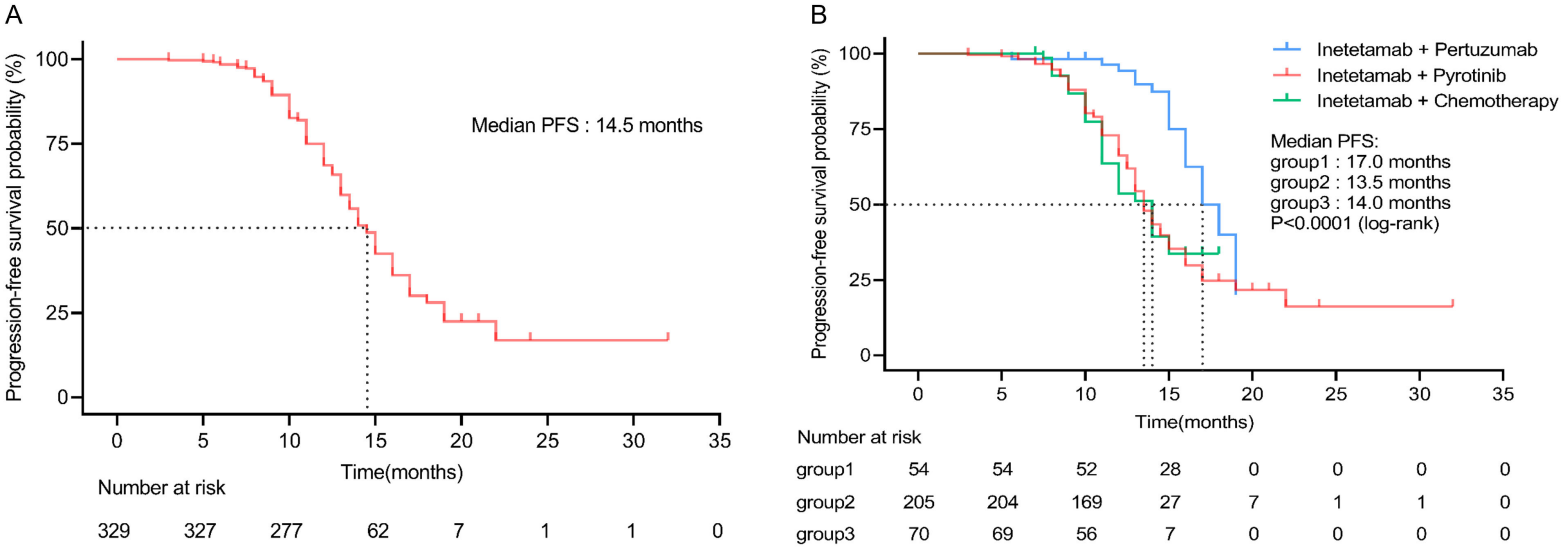
**(A)** Progression-free survival of all patients. **(B)** Progression-free survival of all patients in different inetetamab-containing regimens. (Group 1: inetetamab plus pertuzumab; Group 2: inetetamab plus pyrotinib; Group 3: inetetamab plus chemotherapy).

**Figure 2:**
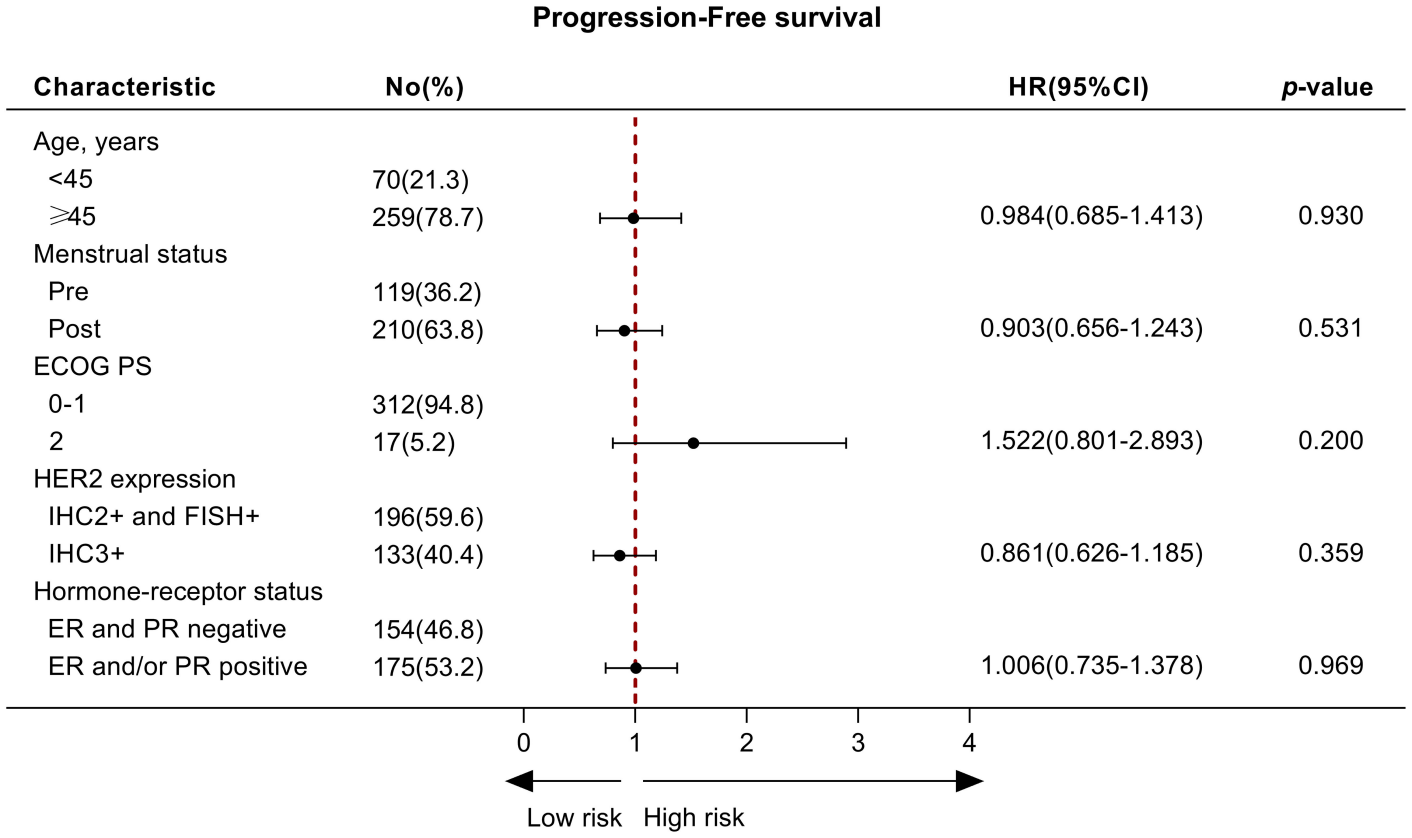
The univariate analysis of factors associated with progression‐free survival. No, number; ER, Estrogen receptor; PR, Progesterone receptor; HER2, Human Epidermal Growth Factor Receptor 2.

Of 329 patients for efficacy evaluation, the median PFS was 14.5 months (**Figure 1**). In addition, we compared the efficacy of the inetetamab-containing regimens in all patients. The median PFS of inetetamab plus pyrotinib was 13.5 months; inetetamab plus chemotherapy was 14.0 months; and inetetamab plus pertuzumab was 17.0 months. There were statistically significant differences among the three groups (*p <*0.001). The estimated PFS rate of inetetamab plus pyrotinib group in first-line at 12 months was 87.6% (95%CI, 79.9%-92.5%), and at 15 months was 42.8% (95%CI, 30.4%-54.6%); in second-line at 12 months was 28.8% (95%CI, 18.1%-40.5%), and at 15 months was 19.4% (95%CI, 9.9%-31.2%), respectively. The PFS rate of inetetamab plus pertuzumab group at 12 months was 94.2% (95%CI, 83.1%-98.1%), and at 15 months was 75.5% (58.6%-86.2%); inetetamab plus chemotherapy group at 12 months was 56.1% (95%CI, 42.2%-67.9%), and at 15 months was 35.2% (95%CI, 18.8%-52.2%), respectively. Univariate analysis revealed that age, menstrual status, ECOG PS, and HR status were uncorrelated with PFS (**Figure 2**).

The CR was observed in 16 (4.9%) patients of all cohort, with the ORR was 51.1% (95% CI, 45.7%-56.4%), and the DCR was 96.4% (95% CI, 93.7%-97.9%). In inetetamab plus pyrotinib group, the ORR was 43.4% (95% CI, 36.8%-50.3%), with 8 (3.9%) patients received CR and 81 (39.5%) received PR. Besides, 115 (56.1%) patients had SD for a DCR of 99.5% (95% CI, 97.3%-99.9%) (**Table 2**). Additionally, 1 (0.5%) patients showed progressive disease (PD).

**Table 2:** Treatment response.

Outcomes	Inetetamab+Pyrotinib Group (n=205)	Inetetamab+Pertuzumab Group (n=54)	Inetetamab+Chemotherapy Group (n=70)
Best response			
Complete response	8 (3.9%)	1 (1.9%)	7 (10.0%)
Partial response	81 (39.5%)	41 (75.9%)	30 (42.9%)
Stable disease	115 (56.1%)	10 (18.5%)	24 (34.3%)
Progressive disease	1 (0.5%)	2 (3.7%)	9 (12.9%)
Objective response rate	89 (43.4%, 36.8%-50.3%)	42 (77.8%, 65.1%-86.8%)	37 (52.9%, 41.3%-64.1%)
Disease control rate	204 (99.5%, 97.3%-99.9%)	52 (96.3%, 87.5%-99.0%)	61 (87.1%, 77.3%-93.1%)

Data are expressed as n (%), n (%; 95% CI).

### 3.3 Subgroup analysis

**Figure 3:**
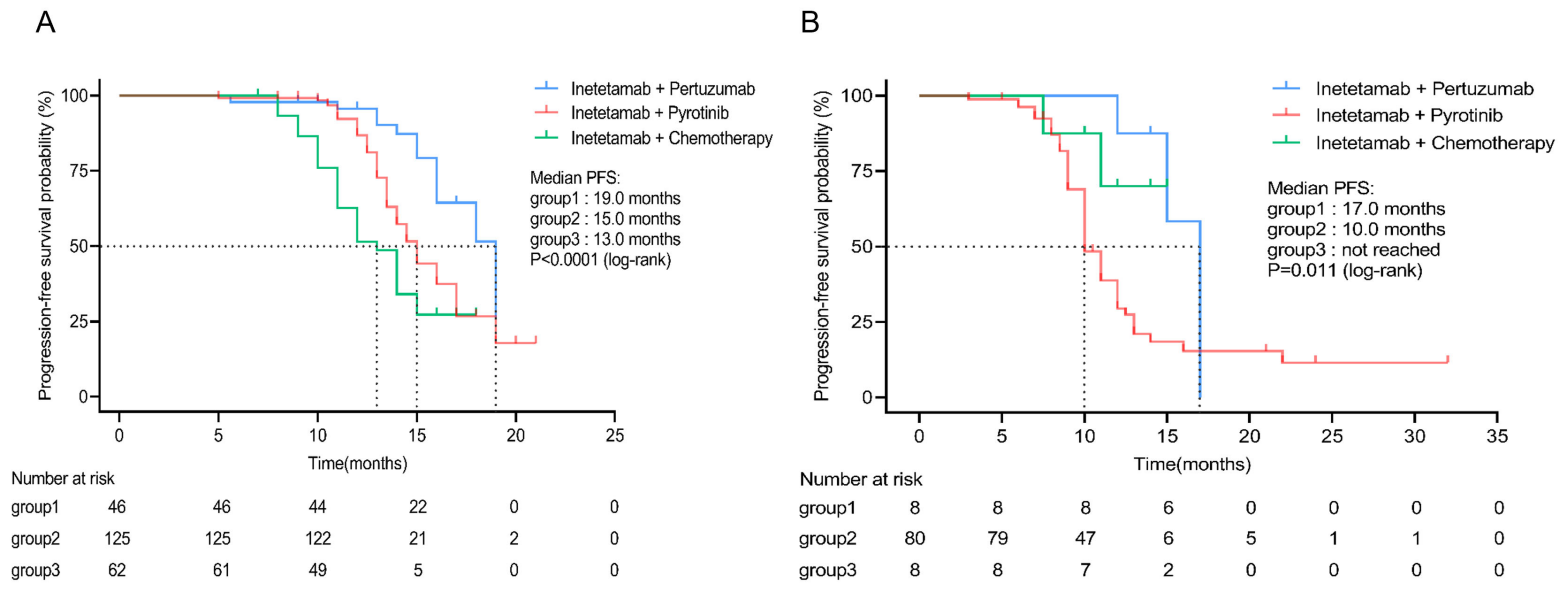
Subgroup analysis of different inetetamab-containing regimens. **(A)** PFS in subgroup patients receiving inetetamab-containing regimens in first line. **(B)** PFS in subgroup patients receiving inetetamab-containing regimens in second line. (Group 1: inetetamab plus pertuzumab; Group 2: inetetamab plus pyrotinib; Group 3: inetetamab plus chemotherapy). PFS, progression-free survival.

The inetetamab plus pertuzumab group had the longest survival compared with inetetamab plus pyrotinib and inetetamab plus chemotherapy group in first-line (mPFS, 19.0 vs. 15.0 vs. 13.0 months) and second-line (mPFS, 17.0 vs. 10.0 vs. not reached months) settings. (**Figure 3**).

### 3.4 Safety and tolerability

The safety were summarized in **Supplementary Table S1**. AEs of any grade occurred in 96.0% of the patients, with diarrhea (176/329, 53.5%), nausea (79/329, 24.0%), and vomiting (76/329, 23.1%) being common. Grade 3 or higher AEs were observed in 29.5% of all 329 patients and mainly included diarrhea (38/329, 11.6%), white blood cell decreased (32/329, 9.7%), myelosuppression (18/329, 5.5%), neutrophil count decreased (7/329, 2.1%). No treatment-related deaths were reported. Compared with the other two subgroups, the incidence AEs of any grade (205/329, 62.3%) and grade ≥3 (80/329, 24.3%) in inetetamab plus pyrotinib group was the highest.

## 4 Discussion

As a trastuzumab biosimilar, inetetamab was approved in the treatment for HER2-positive MBC due to good efficacy and safety in previous clinical trials in China (11, 13, 16, 17), This study further complemented current real-world data and conducted a direct comparison of the clinical practice of inetetamab-containing combination regimens in first-line/second-line treatment of HER2-positive MBC. To the best of our knowledge, this is the largest reported HER2-positive MBC cohort with inetetamab-containing combination regimens at present. In our study, inetetamab-containing regimens demonstrated good efficacy and safety across different treatment regimens. The ORR of the patients in this study was 51.1%, and the DCR was 96.4%, suggesting the strong therapeutic potential of inetetamab in late-line treatment.

The current standard treatments for the HER2-positive MBC include trastuzumab combined with pertuzumab and docetaxel regimen in the first-line setting (18). Furthermore, given good efficacy in previous clinical trials in China, pyrotinib was approved in the second-line treatment for HER2-positive MBC (14, 15, 19). Based on the established benefits of trastuzumab, our study explored the replacement of trastuzumab with a biosimilar inetetamab, aiming to determine whether results comparable to those recommended by clinical guidelines or those obtained in previous trials could be obtained. The CLEOPATRA study showed that the median PFS was 18.7 months of trastuzumab plus pertuzumab combined with docetaxel therapy in HER2-positive MBC patients (20). By contrast, for patients receiving first-line dual-targeted therapy with inetetamab plus pertuzumab, the median PFS reached 19.0 months, which was comparable to CLEOPATRA study. These findings provide a real-world re-confirmation that inetetamab-based dual-targeted regimens may serve as promising alternatives to trastuzumab-containing therapies. Moreover, it is noteworthy that inetetamab-based regimens have potential cost-effectiveness. Although being covered by national health insurance and having experienced substantial price cuts, originator trastuzumab remains more expensive than its biosimilars (21). Higher accessibility and affordability of inetetamab may benefit a larger number of patients, particularly in real-world clinical settings. Despite inetetamab-containing combination showed promising efficacy in second-line (inetetamab plus pertuzumab group: 17.0 months), there is a significant gap compared to the antibody-drug conjugate T-DXd (median PFS=28.8 months) according to the results from DESTINY-Breast03 trial (22). Notwithstanding, high prices of TDM1 and T-DXd results in limitations in the ability to use in clinical practice (13). Thus, considering that the efficacy of our first-line and second-line treatments is comparable to the CLEOPATRA study, inetetamab may be considered as an alternative treatment option.

Certainly, further prospective studies with a larger sample size are necessary to make a definitive conclusion regarding the efficacy of the combination of inetetamab regimen in this population. Generally, clinical trials are conducted under idealized and rigorously controlled conditions, which are not broadly representative of real-world patients and may limit their external validity. In contrast, real-world studies offer a more authentic representation of the clinical landscape, to comprehensively assess the efficacy and safety of inetetamab in relatively short marketing time, thereby enabling better guidance in clinical practice.

Among our patients, inetetamab plus pyrotinib group, inetetamab plus pertuzumab group, and inetetamab plus chemotherapy group were mainly accepted. By contrast, the majority of patients (205/329, 62.3%) in our study chose the inetetamab plus pyrotinib with chemotherapy, with good efficacy and safety (mPFS was 13.4 months). A previous study showed that inetetamab combined with pyrotinib and vinorelbin demonstrated better efficacy than inetetamab combined with pyrotinib alone, indicating that adding chemotherapy to the combination of inetetamab and pyrotinib significantly improved ORR (34.3%) and PFS (7.8 months) (23). In addition, pyrotinib is more easily available and affordable than the other novel anti-HER2 agents. Given this, this is believed to be the primary factor contributing to the widespread adoption of this regimens among a considerable number of individuals. The patients, who received first-line inetetamab plus pyrotinib treatment, had longer mPFS (15.0 months) compared with those who received second-line (10.0 months) in the present study; these results were consistent with those obtained by Li et al. (24). The same status was observed in the other two subgroups. This suggested that the earlier use of inetetamab-containing regimens might be beneficial for the patients. However, it still needs rigorous randomized controlled trials to verify the efficacy of pyrotinib in the first-line treatment of HER2-positive MBC.

Our study also demonstrated that inetetamab combined with pertuzumab might was associated with a longer median PFS among three combination treatments for HER2-positive MBC patients, with a median PFS of 15.1 months and without increasing the incidence of AEs. ADCC is an important mechanism of action for targeted monoclonal antibodies, and modifying the Fc segment to enhance the ADCC effect is important for effectively improving the efficacy of anti-HER2 antibodies (25). Inetetamab had 1.11 times the ADCC effect of trastuzumab, and the strong ADCC effect may be a reason for the good efficacy of inetetamab (26). However, this observation warrants further consideration due to limitations in the sample size of inetetamab plus pertuzumab and individual variations; thus, more research in clinical settings is needed to validate this finding. Previous study suggests that distinct chemotherapy regimens were used for administering HER2 antibodies in clinical practice, which may have an impact on their efficacy (27). Thus, comparing the efficacy of different chemotherapies on the basis of inetetamab in combination with other targeted drugs is an issue worthy of further study.

The current safety profile had no new safety incidents compared to previous inetetamab-containing regimens, and no new safety signals were identified. Most AEs were grade 1-2. The most common grade 3 and higher AEs were similar to previous studies, with inetetamab plus pyrotinib being the predominant occurrence. Among the grade 3 and higher AEs, diarrhea, white blood cell count decreased, and myelosuppression were the most common, potentially attributable to the use of pyrotinib (6, 24, 28). As adverse reactions due to diarrhea accounted for more than half of the AEs reported in this study, continual monitoring for signs of diarrhea during inetetamab-containing treatment should be considered. Notably, cardiac toxicity as a class effect of anti-HER2 therapy, was observed in only <1% of patients in our study, demonstrating the potential advantage cardiac safety profile of inetetamab.

Certain limitations of our study should be acknowledged. First, the selection biases were inevitable due to the nonrandomized and retrospective nature of the study. To reduce retrospective bias, only patients with complete efficacy data were included, and data integrity was manually verified by at least two independent researchers across all centers. Second, the medical records might omit important information about AEs even though we have thoroughly reviewed the patient’s examination results and medical records, such as treatment discontinuation or dose reduction, which resulted in deviations in our results. Undoubtedly, these unresolved critical issues would be emphasized in our future clinical study. Third, the result that the median PFS was not reached in the second-line inetetamab plus chemotherapy group should be interpreted with caution due to the relatively small sample size. Forth, despite no centralized pathology review was performed, each center conducted according to the standardized IHC and FISH analyses. Overall, despite its imperfections, these real-world data may provide insight for testing inetetamab-containing regimens in different treatment lines for HER-2 positive MBC patients in large prospective trials.

## 5 Conclusion

This largest-scale real-world study revealed the efficacy and safety profiles of inetetamab-containing regimens in first-line and second-line in a Chinese MBC patient population to date. In particular, the inetetamab plus pertuzumab in first-line treatment is worth exploring in future clinical trials.

## Data Availability

The original contributions presented in the study are included in the article/**Supplementary Material**. Further inquiries can be directed to the corresponding author.
