# Supplementary material for: Efficacy and safety of inetetamab-containing regimens in patients with HER2-positive metastatic breast cancer in first-line/second-line setting
Source: Front Oncol. 2025 May 29;15:1564888. doi: 10.3389/fonc.2025.1564888 (PMC12158944; doi:10.3389/fonc.2025.1564888)
Supplement: Supplementary file 1 [file Table1.docx]

**Supplement Table S1. Adverse events of patients in inetetamab-containing regimens.**

| **Adverse events** | **Inetetamab+Pyrotinib Group (n=205)** | | **Inetetamab+Pertuzumab Group (n=54)** | | **Inetetamab+ Chemotherapy Group (n=70)** | | **Safety population** **(n=329)** | | |
| --- | --- | --- | --- | --- | --- | --- | --- | --- | --- |
|  | **Any grade** | **Grade ≥3** | **Any grade** | **Grade ≥3** | **Any grade** | **Grade ≥3** | **Any grade** | **Grade ≥3** | |
|  | 205 (62.3%) | 80(24.3%) | 54 (16.4%) | 15 (4.6%) | 57 (17.3%) | 2 (0.6%) | 316 (96.0%) | | 97 (29.5%) |
| Hematologic |  |  |  |  |  |  |  | |  |
| White blood cell count decreased | 64 (19.5%) | 32 (9.7%) | 3 (0.9%) | 0 | 7 (2.1%) | 0 | 74 (22.5%) | | 32 (9.7%) |
| Neutrophil count decreased | 27 (8.2%) | 7 (2.1%) | 20 (6.1%) | 0 | 5 (1.5%) | 0 | 52 (15.8%) | | 7 (2.1%) |
| Myelosuppression | 35 (10.6%) | 17 (5.2%) | 0 | 0 | 5 (1.5%) | 1 (0.3%) | 40 (12.2%) | | 18 (5.5%) |
| Anemia | 34 (10.3%) | 3 (0.9%) | 0 | 0 | 2 (0.6%) | 0 | 36 (10.9%) | | 3 (0.9%) |
| Platelet decreased | 4 (1.2%) | 0 | 2 (0.6%) | 0 | 6 (1.8%) | 0 | 12 (3.6%) | | 0 |
| Hemoglobin decreased | 8 (2.4%) | 0 | 0 | 0 | 0 | 0 | 8 (2.4%) | | 0 |
| Non-hematologic |  |  |  |  |  |  |  | |  |
| Diarrhea | 156 (47.4%) | 29 (8.8%) | 18 (5.5%) | 8 (2.4%) | 2 (0.6%) | 1 (0.3%) | 176 (53.5%) | | 38 (11.6%) |
| Nausea | 70 (21.3%) | 1 (0.3%) | 2 (0.6%) | 0 | 7 (2.1%) | 0 | 79 (24.0%) | | 1 (0.3%) |
| Vomiting | 70 (21.3%) | 1 (0.3%) | 2 (0.6%) | 0 | 4 (1.2%) | 0 | 76 (23.1%) | | 1 (0.3%) |
| Palmar-plantar erythrodysesthesia syndrome | 52 (15.8%) | 1 (0.3%) | 0 | 0 | 0 | 0 | 52 (15.8%) | | 1 (0.3%) |
| Fever | 42 (12.8%) | 1 (0.3%) | 0 | 0 | 0 | 0 | 42 (28.6%) | | 1 (0.3%) |
| Fatigue | 24 (7.3%) | 0 | 2 (0.6%) | 0 | 14 (4.3%) | 0 | 40 (12.2%) | | 0 |
| Appetite loss | 21 (6.4%) | 0 | 1 (0.3%) | 0 | 0 | 0 | 22 (6.7%) | | 0 |
| ALT increased | 15 (4.6%) | 1 (0.3%) | 0 | 0 | 0 | 0 | 15 (4.6%) | | 1 (0.3%) |
| AST increased | 15 (4.6%) | 1 (0.3%) | 0 | 0 | 0 | 0 | 15 (4.6%) | | 1 (0.3%) |
| Alopecia | 6 (1.8%) | 0 | 11 (3.3%) | 0 | 2 (0.6%) | 0 | 19 (5.8%) | | 0 |
| Urticaria | 12 (3.6%) | 0 | 1 (0.3%) | 0 | 0 | 0 | 13 (4.0%) | | 0 |
| Altered GI function | 9 (2.7%) | 2 (0.6%) | 2 (0.6%) | 0 | 1 (0.3%) | 0 | 12 (3.6%) | | 2 (0.6%) |
| Stomatitis | 12 (3.6%) | 0 | 0 | 0 | 0 | 0 | 12 (3.6%) | | 0 |
| Abnormal liver function | 7 (2.1%) | 0 | 0 | 0 | 3 (0.9%) | 0 | 10 (3.0%) | | 0 |
| Weight loss | 7 (2.1%) | 0 | 0 | 0 | 0 | 0 | 7 (2.1%) | | 0 |
| Dizziness | 3 (0.9%) | 0 | 0 | 0 | 3 (0.9%) | 0 | 6 (1.8%) | | 0 |
| Upper respiratory infection | 5 (1.5%) | 0 | 0 | 0 | 0 | 0 | 5 (1.5%) | | 0 |

Note: Data are expressed as n (%).

Abbreviation: ALT, Alanine aminotransferase; AST, Aspartate aminotransferase; GI, Gastrointestinal.
